# A Web-Based Self-assessment Model for Evaluating Multidisciplinary Cancer Teams in Spain: Development and Validation Pilot Study

**DOI:** 10.2196/29063

**Published:** 2022-03-10

**Authors:** Mercedes Guilabert, Joan Prades, Josep M Borras, Inmaculada Maestu, Juan Antonio Guerra, Lluís Fumadó, José Joaquin Mira, AEMAC Program Research Team

**Affiliations:** 1 Health Psychology Department Miguel Hernandez University Elche Spain; 2 Department of Health Catalonian Cancer Strategy Barcelona Spain; 3 Department of Clinical Sciences University of Barcelona Instituto de Investigación Biomédica de Bellvitge Barcelona Spain; 4 Medical Oncology Service Doctor Peset University Hospital Valencia Spain; 5 Department of Hematology and Oncology Fuenlabrada University Hospital Fuenlabrada Spain; 6 Urological Cancer Functional Unit Hospital del Mar-Parc de Salut Mar-IMIM Barcelona Spain; 7 Atenea Research Group Foundation for the Promotion of Health and Biomedical Research Sant Joan d’Alacant Spain; 8 Alicante-Sant Joan Health Department Alicante Spain; 9 see Authors' Contributions

**Keywords:** web-based tool, multidisciplinary care, cancer, evaluation, quality assurance

## Abstract

**Background:**

Tumor boards constitute the main consensus and clinical decision–making body of multidisciplinary teams (MDTs) in cancer care. With the increasing clinical complexity of treatment options (eg, targeted therapies, multimodal treatments) and the progressive incorporation of new areas of intervention (eg, survivorship care), tumor boards are now required to play a central role in all cancer processes. However, although frameworks are in place to evaluate MDT quality, only few web-based tools are available for this purpose; indeed, no web-based MDT evaluation tools have been developed for or adapted to the Spanish National Health System.

**Objective:**

The first aim of this study was to develop a web-based self-assessment model (Autoevaluación de Equipos Multidisciplinares de Atención al Cáncer [AEMAC]) for evaluating multidisciplinary cancer teams in Spain and the second aim was to validate this tool by testing its metric properties, acceptability, and usability.

**Methods:**

We designed and validated the AEMAC program in 3 stages. In the first stage (research), we reviewed the available scientific evidence and performed a qualitative case study of good practice in multidisciplinary care within the Spanish National Health System (n=4 centers and 28 health care professionals). The results were used to define the thematic areas and quality criteria for the self-evaluation model, which were then discussed and validated by a group of experts. The second stage (development) involved the technological development of a web app that would be accessible from any mobile device. In the third stage (piloting and validation), we conducted 4 pilot tests (n=15 tumor boards, 243 professionals) and used the results to analyze the acceptability and usefulness of the tool.

**Results:**

We designed a self-assessment model based on 5 thematic areas encompassing a total of 25 quality components, which users rated on a 3-option development scale. The evaluation process, which was managed entirely from the web app, consisted of individual self-assessment, group prioritization, and creation of an improvement plan. Cronbach alpha (.86), McDonald’s omega (0.88), and various fit indices (comparative fit index between 0.95 and 1 and goodness-of-fit index between 0.97 and 0.99 for all 5 aspects) confirmed internal consistency. The mean rating for overall satisfaction with the tool and for consistency between the content of the tool and the reality of tumor boards was 7.6 out of 10.

**Conclusions:**

The results obtained during the period of research and piloting of the AEMAC program showed that it has an appropriate structure and metric properties and could therefore be implemented in a real context and generalized to other hospitals. As a virtual tool, it helps to measure the key aspects of MDT quality, such as effectiveness of collaboration and communication, leadership, and the organizational environment.

## Introduction

### Background

Tumor boards constitute the main consensus and clinical decision–making body of multidisciplinary teams (MDTs) in cancer care [1]. The direct and indirect benefits of MDTs have been confirmed through extensive analysis [2-8], and the proper organization and systematic implementation of these bodies has become the central focus of cancer control policies [9]. With the increasing clinical complexity of treatment options (eg, targeted therapies, multimodal treatments) and the progressive incorporation of new areas of intervention such as attention to survival as well as health care objectives such as the reduction of waiting times, MDTs have come to assume a central role in cancer care [10].

### Rationale of MDT Assessment

Tumor boards are generally based on regular work sessions that require the participation of a large number of professionals and departments, but hospitals often fail to define the internal organization of these boards, the scope of their work, or the resources needed. Tumor boards should be subject to analysis and intervention based on their importance for patients and the economic costs involved to assess their degree of usefulness and the quality of their decisions and to establish necessary changes. From this perspective, the multiple benefits they may provide do not inevitably result from a policy decision [11]. In the context of the Spanish National Health System (SNS), although the cancer strategy set out by the Ministry of Health defines MDTs as a reference framework for oncology services [12], the degree of MDT formalization and work capacity varies substantially between hospitals [13,14]. Moreover, although many scientific societies and European organizations have made attempts to strengthen MDTs by establishing quality criteria and work procedures, only few web-based tools are in place for evaluating the effectiveness of teamwork. The tools currently available, such as MDT-FIT (MDT-feedback for improving teamworking) [15], MDT-OARS (MDT-observational assessment rating scale) [16,17], MDT-QuIC (MDT-quality improvement checklist) [18], and MDT-MODe (MDT-metric of decision making) [19] vary in terms of methodology (eg, checklist, observation) and availability as a virtual tool [20].

### Routines and Gaps in the Work of Tumor Boards in Spain

The large number of professionals involved in cancer care and the complexity of the diagnostic therapeutic process mean that coordination problems and poor communication are very likely to occur. The evidence points to the importance of these aspects, which can contribute to improving the survival and quality of life of patients with cancer. Thus, the implementation of a developed model of multidisciplinary care is one of the greatest challenges to progress in improving the quality of care for patients with cancer. In Spain, within the context of the SNS, the complexity of multidisciplinary care and its impact on cancer care depends on many factors: the different organizational forms according to the territories, decision making in the diagnostic and therapeutic process, or the cultural resistance to change that working in a multidisciplinary manner may entail. Since there are many barriers to the implementation of multidisciplinary care, this interactive tool can enable the diagnosis of MDTs, thereby offering a map of interventions for the proper functioning of the teams. Other international experiences [15-19] highlight the fact of having an instrument to perform self-diagnosis; the tool presented here additionally makes it possible to have the tool available in a web-based format, which facilitates the rapid creation of a roadmap for the appropriate management of the tumor committee within the SNS.

### Autoevaluación de Equipos Multidisciplinares de Atención al Cáncer Program

To promote multidisciplinary cancer care within the SNS, we developed a program for the self-assessment of MDTs in cancer care (AEMAC, by its acronym in Spanish [Autoevaluación de Equipos Multidisciplinares de Atención al Cáncer]). The AEMAC program is a web-based tool designed to facilitate the evaluation of critical components related to the internal structure, coordination and communication, organizational context, and development possibilities of MDTs. The objective of this study was to research, develop, and validate the AEMAC program as a quality self-assessment tool for MDTs.

## Methods

This study was performed in 3 stages.

### Research and Expert Assessment

The first stage, focused on the design of the tool, drew on 2 sources of information: the available scientific literature (including gray literature) and the experiences of tumor boards from hospitals of varying structure, size, and capacity to attend patients with cancer.

First, we updated a systematic review that had been conducted in 2014 by 2 of the authors of this paper (JP and JMB), extending the search to November 2016 while maintaining the same search criteria [10]. We also consulted a set of strategic documents to analyze organizational principles and standards in multidisciplinary care [17,21-27]. We decided to base the first version of the AEMAC program on the document *Policy Statement on multidisciplinary cancer care* [21], which defines the core elements that all tumor-based MDTs should include. By synthesizing the information collected in this systematic review, we were able to draft the interview script to be used in the development stage.

Second, we performed a qualitative case study on practices of excellence in multidisciplinary care in Spanish public hospitals in the period spanning May 2017 to September 2017 [28,29]. We included tumor boards specializing in specific tumors of all prognoses and locations and more general boards (eg, digestive system tumors) with a structured MDT that had been operating for at least 3 years in SNS hospitals located in all regions of Spain. A further inclusion criterion was the presence of a clinical unit for centralizing the whole care process of specific pathologies. After the selection process, we planned on-site visits to study in detail the work of MDTs in the following hospitals: Hospital Universitario del Mar, Barcelona (lung cancer); Hospital Universitario Ramón y Cajal, Madrid (breast cancer); Complejo Hospitalario de Navarra (digestive system tumors); and Hospital Clínico de Salamanca (multiple cancers). This sample of hospitals was endorsed by the scientific societies involved in the program (Table S1 of Multimedia Appendix 1). Additionally, we interviewed 20 professionals from the following specialties: medical oncology (n=4), radiation oncology (n=4), other medical-surgical specialties (n=4), pathological anatomy (n=2), radiology (n=4), and nursing (n=2). These visits and interviews were aimed at identifying the key thematic areas and components to include in the MDT self-assessment model.

The resulting preliminary matrix was assessed by 6 SNS experts who were selected according to the criteria of territorial representativeness, different specialties, levels of care, and scientific or management experience (as of September 2017). Specifically, they assessed the readability, face validity, and content of the tool according to the legibility of the identified components and coherence of the model and its elements; the adaptability of the components to the reality of the SNS; the weighting of the components according to their level of importance; and the definition of future components.

### Information Technology Development and Phases in the Self-assessment Process

**Figure 1:**
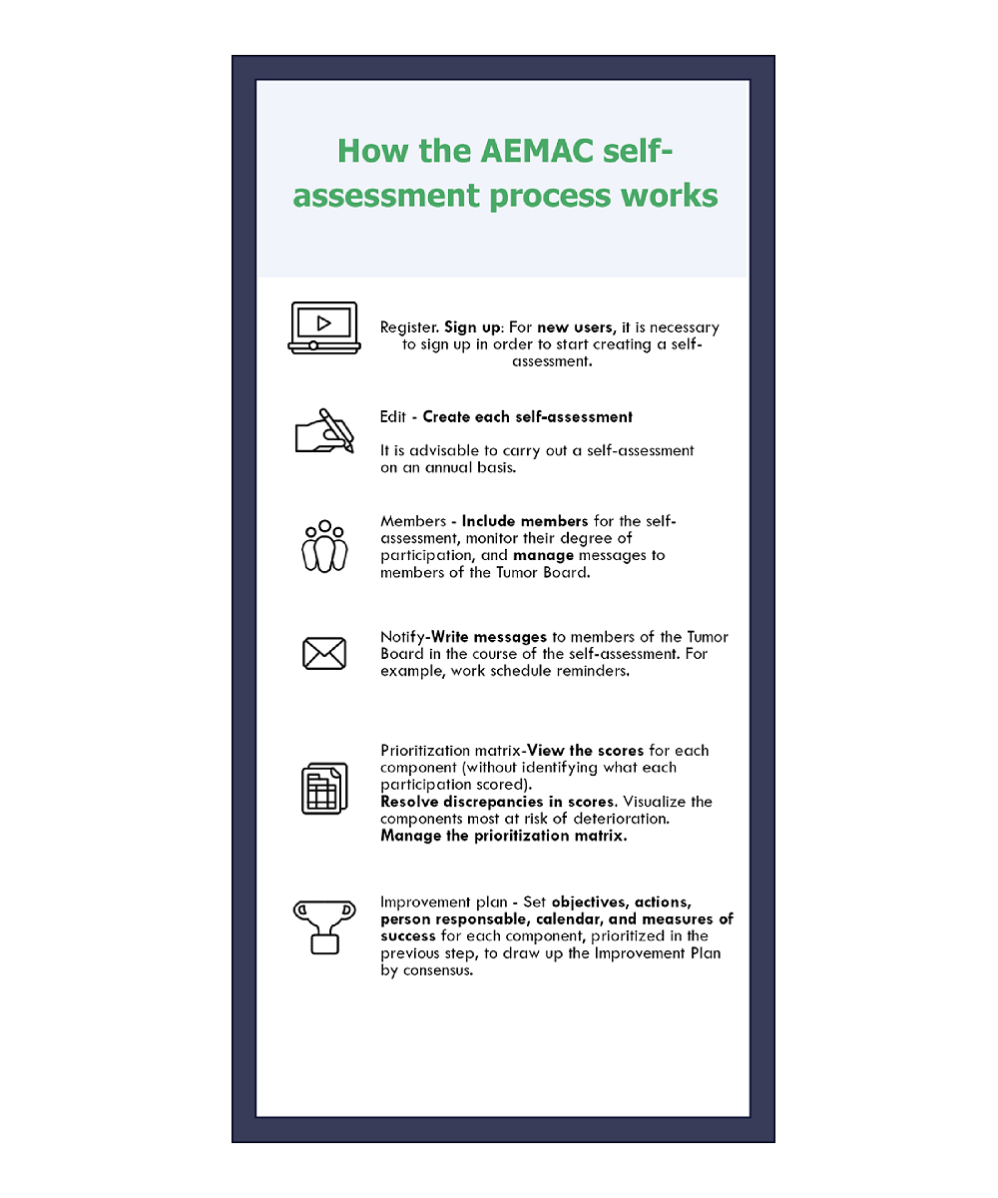
Working of the AEMAC self-assessment process. AEMAC: Autoevaluación de Equipos Multidisciplinares de Atención al Cáncer.

The second stage consisted of developing the web-based tool. The AEMAC program was conceived from the beginning as a web app that could be activated and managed from any mobile device. The web-based tool [30] was designed to facilitate consensus within the tumor board on areas for improvement and possible interventions and to establish the work schedule for achieving these improvements and ensuring their sustainability. The self-assessment was structured in 3 phases (see Textbox 1, Multimedia Appendices 2-4, and Figure 1).

**Phases in the Autoevaluación de Equipos Multidisciplinares de Atención al Cáncer program self-assessment process.**

**Phase 1: Individual self-assessment (Multimedia Appendix 2)**

- All board members independently fill out the web-based questionnaire.
- Each component has 3 evaluation possibilities, ranging from least development (first option) to greatest development (third option), with an intermediate category (second option).
- The evaluation process for each component is completed with a subquestion on the “possibility of improvement” if option 1 is chosen or on the “risk of worsening” if option 3 is selected. These items are evaluated based on a scale of 0 to 10 points, and this score is included in the prioritization algorithm. For example, components with poor ratings and without the possibility of improvement obtain a lower relative weight.

**Phase 2: Group prioritization process (Multimedia Appendix 3)**

- The team (or a subgroup of 3 team members) assesses the results, resolves any team discrepancies that emerged in phase 1, and prioritizes areas for improvement.
- Discrepancies occur when none of the 3 response options accounts for at least 60% of the responses for any component.

**Phase 3: Creating an improvement plan (Multimedia Appendix 4)**

- The team sets objectives and actions for an improvement plan spanning 1 to 2 years.
- Responsible parties are assigned and indicators are established so that the degree of achievement can be measured.

Data are stored and participants’ identities are protected on the server at Miguel Hernández Public University to ensure anonymous data management. This is fundamental because the AEMAC program automatically shares compared information with other MDTs of the same hospital level and pathology to enhance benchmarking. The hospital level is captured through the postal code of the hospital that was provided during the initial registration of the self-assessment process.

### Piloting and Validation

The third stage included a pilot study to test the adequacy of the procedure and to analyze the metric characteristics of the developed instrument. To test the acceptability and usefulness of the AEMAC program, pilot tests were performed in 4 SNS hospitals—Hospital Universitario del Mar (Barcelona), Hospital Universitario de Fuenlabrada (Madrid), Hospital Universitario Doctor Peset (Valencia), and Hospital Universitario La Paz (Madrid)—including a total of 15 tumor boards and 243 professionals. These hospitals belonged to 3 different regional health services and were representative of the different types of Spanish public hospitals. The number of beds ranged from 400 to 1000. Three researchers attended these meetings as observers, information technology consultants, and evaluators of the experience (JJM, MG, JP).

The first main objective of the pilot tests was to observe the clarity of the concepts used in each component and the consistency of the criteria used to define these concepts. These criteria include correlation (ie, if the tumor board hypothesized that good multidisciplinary work was being done in different areas, the self-assessment of the professionals on the tumor board should be in segment 3 of the areas they considered to be strengths), variability (ie, the second option should not be selected by default), and acceptability (ie, professionals should not feel penalized by “negative” language). The second aim of the pilot tests was to assess the usability and functionality of the website, considered as critical elements in the time for individual completion of the questionnaire and the general usability of the instrument (eg, interface user friendliness, ease of transition between phases). Additionally, the responses to each of the components were analyzed considering both floor and ceiling effects to rule out elements with little variability, elements that could not be implemented, and elements that were already fully implemented.

We also analyzed the degree of discrepancy in the evaluations of different members of the tumor boards for each component, acknowledging that systematic discrepancies between all participants of all boards must represent inappropriate wording. Finally, through confirmatory factor analysis, the coherence of the grouping of components by thematic area was analyzed using the comparative fit index, Jöreskog and Sörbom’s goodness-of-fit index, adjusted goodness-of-fit index, root mean square residual, standardized root mean square residual, and root mean square error of approximation. The model was accepted when comparative fit index, Jöreskog and Sörbom’s goodness-of-fit index, and adjusted goodness-of-fit index scores were greater than 0.95 and root mean square residual, standardized root mean square residual, and root mean square error of approximation scores were less than 0.08 [31,32]. The internal consistency of the resulting factorial solution was analyzed using Cronbach alpha and McDonald’s omega statistical indicators.

A total of 243 pilot study participants were asked to complete a survey to assess the critical issues of the tool (Multimedia Appendix 5). The anonymized responses were analyzed using descriptive statistics. The first part of the survey was designed to assess user experience of the AEMAC program and included 5 items with a response scale ranging from 0 (unsatisfactory) to 10 (totally satisfactory). The questions covered ease of answering the AEMAC questionnaire, coherence between questions, adequacy of the response options, and the method for establishing an improvement plan. The second part of the survey aimed to evaluate the adequacy of the established critical and semicritical components.

### Ethics Approval

The study was approved by the Ethics and Research Integrity Committee of the Miguel Hernández University (AUT.DPS.JMS.02.21).

## Results

### Research and Expert Assessment

By the end of the research stage, we were able to define the following 5 thematic areas: (1) preparation and organization of the tumor board, (2) board decision-making process, (3) continuity of care, (4) organizational context, and (5) cross-disciplinary roles and team cohesion. These 5 thematic areas encompassed a total of 27 components considered to have a potential impact on quality of care. The following changes were made following the expert assessment of the preliminary matrix:

1. *Legibility of the identified components and coherence of the model and its elements*. Two components were added, and changes were made in the wordings to improve clarity and relevance and to avoid any overlaps.

2. *Adaptability of the components to the reality of the SNS*. It was decided to ensure adaptability of the AEMAC program to any MDT, regardless of hospital level (university, community, or local hospital).

3. *Weighting of the components according to their level of importance*. The experts agreed on different weights for the components according to the categories “normal,” “high,” or “very high” importance. This had an impact on the prioritization algorithm used during the self-assessment.

4. *Definition of future components.* Six components were defined with the prospect of being included in 5 years to replace outdated components.

The adjusted matrix can be seen in Table S2 of Multimedia Appendix 1.

### Information Technology Development and Phases in the Self-assessment Process

The AEMAC program was developed as a 3-phase process with the dual objective of gathering individual and aggregated perspectives on the quality of teamwork and encouraging dialogue, negotiation, and formulation of possibilities for improvement based on the critical aspects identified.

### Piloting and Validation

The pilot study participants agreed that the website format and the fact that the questionnaire could be completed on any mobile device made the tool easy to use. Discrepancies could be resolved by a subgroup of professionals, which facilitated the process, although some teams had reservations in this regard. When faced with certain questions, some professionals stated that they required more information to give their opinion, usually because of lack of direct contact with patients. We, nonetheless, agreed to maintain the obligation of answering all the standards included in the self-assessment questionnaire. The time taken to complete the AEMAC questionnaire ranged from 12 to 14 minutes, which was considered satisfactory. The average group discussion time was 15 minutes for phase 2 (range, 10-20 minutes) and 30 minutes for phase 3 (range, 20-50 minutes). The aspect with the lowest acceptability rating was the registration process for initiating a self-assessment. MDTs in the SNS usually have no administrative support, which means registration has to be managed by the professionals themselves.

For most components of the thematic areas “Preparation and organization of the board” and “Continuation of the care process,” the third option (“Representing greater development”) was selected more frequently than either of the other two. Regarding “Board decision-making process” and “Organizational context,” the bulk of the responses fell between options 1 and 2. Finally, great variability in the responses was observed in “Cross-disciplinary roles and team cohesion”. Table S3 of Multimedia Appendix 1 shows the combined scores from all boards, indicating the range of scores observed and the response frequencies of each option for each component. The results of this first analysis identified no ceiling or floor effects to substantiate eliminating any of the 27 components. However, after reviewing the confirmatory factor analysis results, we eliminated the components “Patient information process” (from the thematic area “Board decision-making process”) and “Team-patient communication framework” (from “Cross-disciplinary roles and team cohesion”). We also moved the “Team cohesion” component to the thematic area “Board Preparation and Organization.” The fit indices of this model confirmed that the various components were well assigned to the thematic areas. Table 1 shows the goodness-of-fit indices for each thematic area of the resulting model. The “Board decision-making process” obtained the best fit indices according to the previously defined benchmark.

**Table 1:** Confirmatory factor analysis adjustment indices.

Adjustment indices	Preparation/organization	Decision making process	Continuity of care	Organizational context	Cross-disciplinary roles
Comparative fit index^a^	0.95	1.00	0.96	0.96	0.99
Jöreskog and Sörbom’s adjusted goodness-of-fit index^b^	0.94	0.99	0.97	0.94	0.98
Jöreskog and Sörbom’s goodness-of-fit index^b^	0.97	0.99	0.99	0.98	0.99
Root mean square residual^c^	0.02	0.01	0.01	0.02	0.01
Standardized root mean square residual^c^	0.05	0.02	0.03	0.04	0.02
Root mean square error of approximation (90% CI)	0.05 (0-0.08)	0 (0-0.1)	0.04 (0-0.1)	0.08 (0.03-0.1)	0.03 (0-0.1)

^a^Ranges from 0 to 1, with a value of 0.9 being the minimum required to defend the model.

^b^Goodness-of-fit index and adjusted goodness-of-fit index: range between 0 and 1 and those models that exceed 0.9 are considered adequate models.

^c^Root mean square error of approximation and standardized root mean square: indicators of a good fit with values less than 0.05.

**Figure 2:**
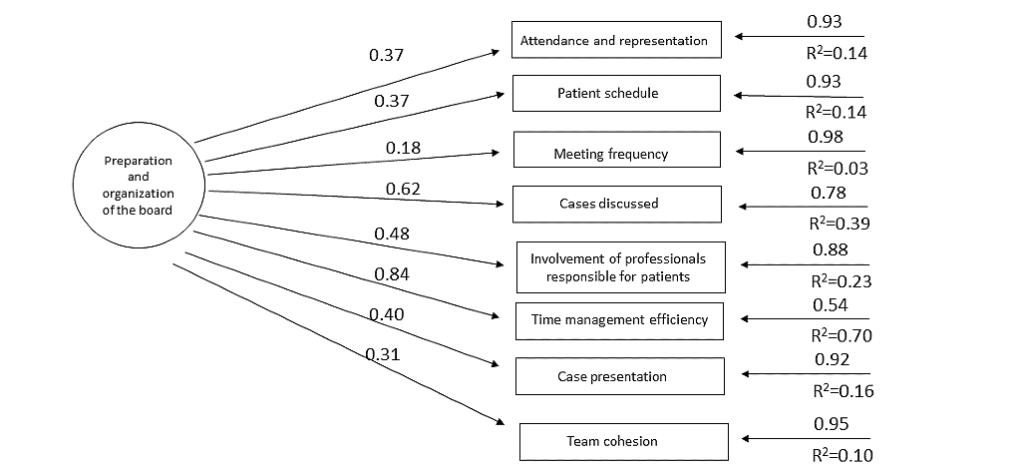
Optimized factor 1 model based on the confirmatory factor analysis of the validation study carried out. The values above the lines are the estimates of the regression coefficients of the common factors and of the specific factors, that is, the contribution of each item to the factors, which is also called factor loadings. The R^2^ is the variance explained by the factor for each of the items.

**Figure 3:**
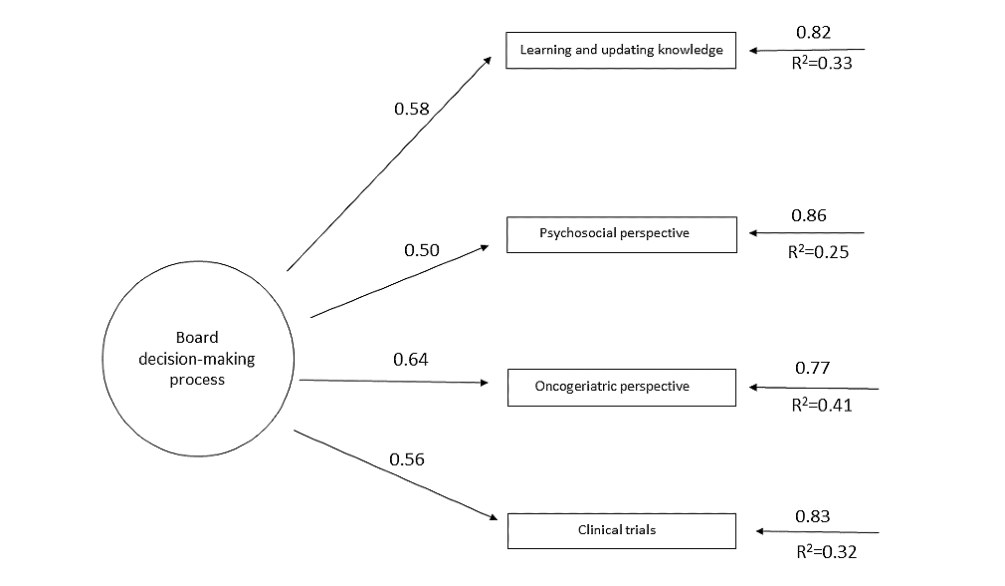
Optimized factor 2 model based on the confirmatory factor analysis of the validation study carried out. The values above the lines are the estimates of the regression coefficients of the common factors and of the specific factors, that is, the contribution of each item to the factors, which is also called factor loadings. The R^2^ is the variance explained by the factor for each of the items.

**Figure 4:**
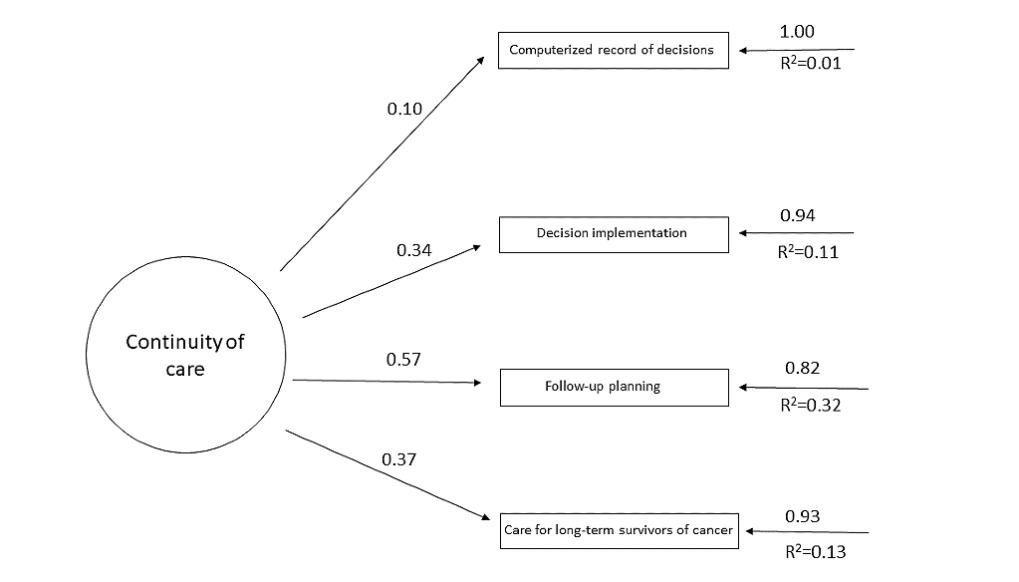
Optimized factor 3 model based on the confirmatory factor analysis of the validation study carried out. The values above the lines are the estimates of the regression coefficients of the common factors and of the specific factors, that is, the contribution of each item to the factors, which is also called factor loadings. The R^2^ is the variance explained by the factor for each of the items.

**Figure 5:**
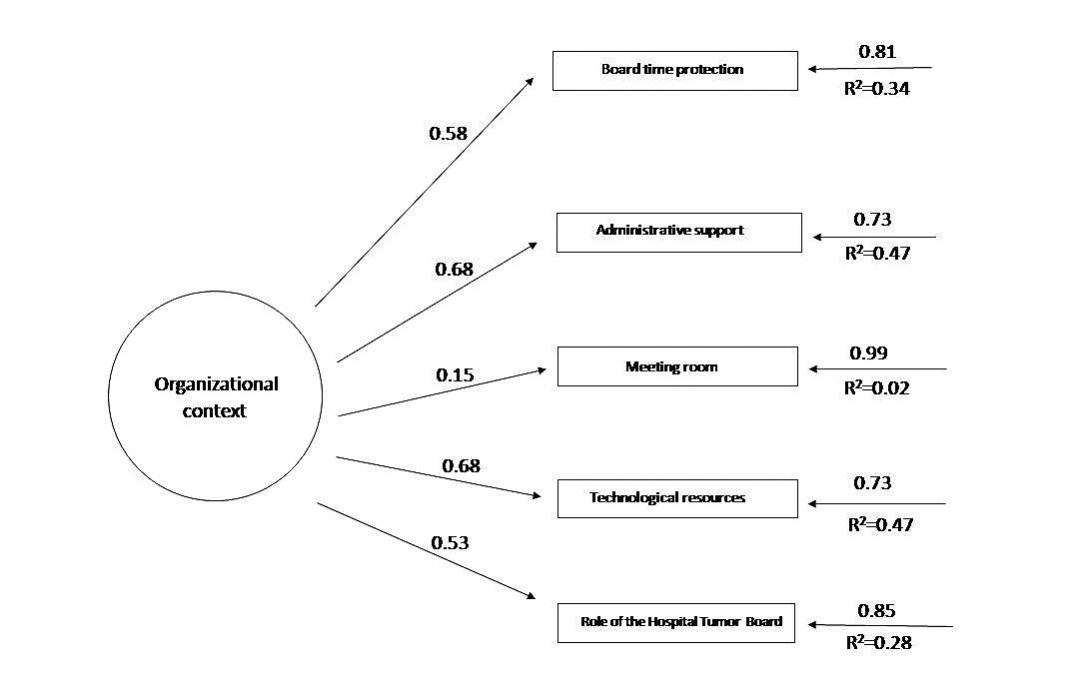
Optimized factor 4 model based on the confirmatory factor analysis of the validation study carried out. The values above the lines are the estimates of the regression coefficients of the common factors and of the specific factors, that is, the contribution of each item to the factors, which is also called factor loadings. The R^2^ is the variance explained by the factor for each of the items.

**Figure 6:**
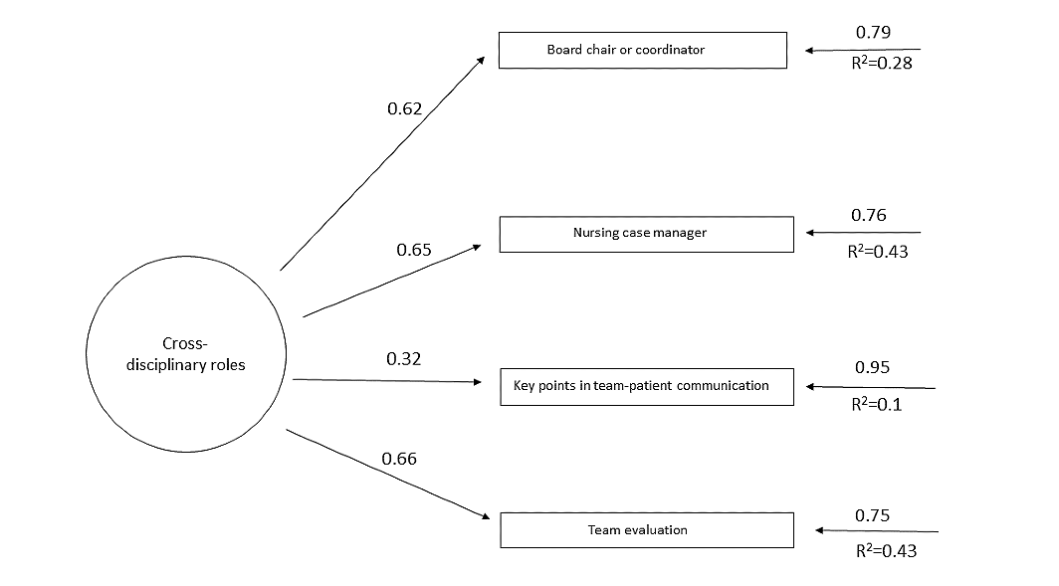
Optimized factor 5 model based on the confirmatory factor analysis of the validation study carried out. The values above the lines are the estimates of the regression coefficients of the common factors and of the specific factors, that is, the contribution of each item to the factors, which is also called factor loadings. The R^2^ is the variance explained by the factor for each of the items.

Table 2 shows the revised structure of the AEMAC program. The optimized model can be seen in Figures 2-6 (optimized model of the questionnaire factorial structure based on confirmatory factor analysis from the validation study performed for each factor, factors 1-5, Structural Equation Modelling Path Diagram chart). The values shown in Figures 2-6 represent the standardized solution of the confirmatory factor analysis equations. The values above the lines in the figures are the estimates of the regression coefficients of the common factors and of the specific factors, that is, the contribution of each item to the factors, which is also called factor loadings. The R^2^ is the variance explained by the factor for each of the items.

In the internal consistency analysis, the AEMAC program achieved a Cronbach alpha score of .86 and a McDonald’s omega score of 0.88. Table 3 and Table 4 show the combined responses of the survey evaluating the AEMAC program. In total, 40 out of 243 professionals responded (16.5% response rate). Seven (88%) of the 8 components that were considered critical and all 3 components that were considered semicritical received a high adequacy rating.

**Table 2:** Structure of the revised Autoevaluación de Equipos Multidisciplinares de Atención al Cáncer program following confirmatory factor analysis.

Thematic area	Items
Preparation and organization of the board	Attendance and representation Patient schedule Meeting frequency Cases discussed Involvement of professionals responsible for patients Time management efficiency Case presentation Team cohesion
Board decision-making process	Learning and updating knowledge Psychosocial perspective Oncogeriatric perspective Clinical trials
Continuity of care	Computerized record of decisions Decision implementation Follow-up planning Care for long-term survivors of cancer
Organizational context	Board time protection Administrative support Meeting room Technological resources Role of the Hospital Tumor Board
Cross-disciplinary roles	Board chair or coordinator Nursing case manager Key points in team-patient communication Team evaluation

**Table 3:** Results of the responses to the satisfaction survey (part 1).

Item	Median score	Minimum score	Maximum score
Ease of answering the questions	8	3	10
Coherence between content of questions and reality of tumor boards	8	3	10
Adequacy of the 3-option response scale	8	3	10
Ease of creating an improvement plan in the web-based app	7	3	10
Overall satisfaction with the self-assessment	8	2	10

**Table 4:** Results of the responses to the satisfaction survey (part 2).

Component		Adequate (N=40), n (%)	Not adequate (N=40), n (%)
**Critical components**			
	Attendance and representation	40 (100)	0 (0)
	Patient schedule	39 (98)	1 (3)
	Learning and updating knowledge	39 (98)	1 (3)
	Decision implementation	38 (95)	2 (5)
	Follow-up planning	35 (88)	5 (12)
	Board time protection	37 (93)	3 (7)
	Board chair or coordinator	39 (98)	1 (2)
	Nursing case manager	23 (59)	16 (41)
**Semicritical components**			
	Cases discussed	34 (95)	6 (5)
	Patient information process	26 (73)	14 (28)
	Computerized record of decisions	33 (92)	7 (8)
	Key points in team-patient communication	27 (77)	13 (23)

## Discussion

### Overview of This Study

The AEMAC program is a web-based tool designed to facilitate the self-assessment of multidisciplinary cancer care teams with a view of improving their internal organization and scope of care. By analyzing the internal and external processes that frame the work of MDTs, we were able to identify 27 critical components that are implemented to a varying degree in the context of the SNS, as well as establish a range of possibilities for the intervention. When the AEMAC program was tested by 15 MDTs, it obtained good results on the content, acceptability, and time required, and showed reasonable psychometric properties, including internal consistency and item discrimination. The perspective acquired during the research process, which included content validation by experts, and the consistency of the results obtained in the pilot study show that the AEMAC program could be implemented in a real context.

### Framework of This Study

In accordance with other authors, we consider that tumor boards stimulate the knowledge and development of the professionals involved in the care of patients with cancer, thereby encouraging teamwork and improving the quality of health care [33,34]. In this regard, several government-produced documents defining MDTs such as the principles of multidisciplinary care in Australia [23], multidisciplinary cancer conferences in the Canadian province of Ontario [22], and the characteristics of an effective MDT in the United Kingdom [17] highlight the importance of internal cohesion and positive leadership styles. The desired consensus of a tumor board should not mask the valuable technical perspectives of its members nor should it be vulnerable to what has been called ego-based medicine [35].

The rationale and health care objectives of the AEMAC program multidisciplinary care model are based on the principles and priorities set out in the reference document *Policy Statement on multidisciplinary cancer care* [21]. For example, one component that reflects a well-developed tumor board according to the AEMAC program is that all cases treated in the hospital for a specific pathology are presented at the board meeting. While this topic “all cases discussed” has some controversy, many health professionals consider that this measure simply implies ensuring evidence-based decisions. European initiatives such as the European Reference Networks [36] share the view of securing evidence-based opinions, which is also reflected in the criteria and standards of various European cancer organizations [24-27]. These bodies are raising awareness in the SNS of the need to have well-structured and well-equipped MDTs that are answerable to a national health authority. The available evidence suggests that significant variability exists between MDTs in terms of objectives, roles, organizational implications, performance, and access [37,38]. In the context of the SNS, these differences are accentuated by considerable decentralization in health care [13].

### Comparisons With Other Instruments and Tools

The AEMAC program follows the inspiring principles of instruments such as the MDT-FIT [15]. In this case, the 3-stage process to perform the self-assessment of the MDTs and the availability of a web-based tool to perform the process have been elements that the AEMAC program has taken into account in the development of the self-assessment process, wherein the time needed to perform the whole process was 10-12 weeks, while the AEMAC program considers that the whole process can be performed in the same morning, leaving a sufficient time interval to perform the process of implementing the Improvement Plan (approximately 6-12 months). MDT-OARS [16,17], which is supposed to be a development and validation model to be followed in the case of the AEMAC program, consists of 47 items and a 5-point scale and is an instrument that each tumor committee must apply. In the case of MDT-QuIC [18], the instrument applies more like a checklist, and in its development study, the attitudes of the people who used it were assessed. The AEMAC program followed this model to assess the experiences of the people who used it. MDT-MODe [19] is based on an observational evaluation tool, where 2 professionals evaluate the behavior of the MDT. The AEMAC program is based on the individual evaluation of each of the committee members in the first phase and then sharing of the results achieved and the areas of top priority.

### Strengths and Weaknesses

#### Strengths of the AEMAC Program

Implementing the AEMAC program could benefit clinicians, managers, and patients. For clinicians, the program not only enables comparison of tangible aspects such as technological resources (eg, use of videoconferencing) but also takes the human factor into account. Elements related to communication style or cooperation (eg, trust, implementation of decisions) are critical but tend to be ignored in evaluations or accreditation systems. For managers at all levels of the SNS, the AEMAC program offers guidance on implementing and developing an effective organizational ecosystem. In Spain, MDTs are not incorporated into the care pathway through mandatory criteria as part of health policy, as they are in the United Kingdom. The AEMAC program facilitates assessment of MDT uniformity in this context. Finally, although the defined model is for professional use, patients stand to benefit to the extent that the quality criteria include the consideration of oncogeriatric and psychosocial aspects, personalization of the information delivered to patients, and proactive organization of patients’ agendas.

#### Weaknesses of the AEMAC Program

Although most evaluations of the content and functionality of the AEMAC program were positive, 80% of the professionals who were asked to evaluate the tool were unable to because of scheduling or other problems. We did not manage to obtain this information subsequently, although we contacted these team members individually after the on-site visits. The main feedback on the experience with the tool was provided by the people who had responsibility for the tumor board (chair and secretary). Given the uniqueness of the AEMAC questionnaire, it was validated through confirmatory factor analysis. Similarly, the difficulty of accessing teams and activating self-assessment processes during the pilot test—mainly due to the gatekeepers being informal contacts or the health professionals showing inconsistent interest in participating—meant that we were unable to include a larger sample of tumor boards. Another limitation was that the boards of only 4 hospitals participated in the pilot tests. These hospitals were similar to a majority of Spanish hospitals: accreditation for teaching, residents, students, and number of beds. As a result, adaptability issues may arise if the program is rolled out in a real context, as the AEMAC program is designed for any MDT of the SNS, and the research process and piloting covered only a few regions of Spain. If the AEMAC program is to become a reference tool, it must be promoted not only by scientific societies but also by the health authorities of each region.

### Future Developments

As MDT work dynamics and structures evolve, future evaluations will determine which elements should be replaced. The AEMAC program is a dynamic quality model based on the improvement cycle that must periodically adapt to reality. The elements incorporated in the future will follow the same principle.

### Conclusion

The AEMAC program is the first web-based quality self-assessment tool for evaluating MDTs in the SNS. The results obtained during the research and piloting period suggest that it could be implemented in a real context. Consensus and multidisciplinary work undoubtedly contribute to clinical effectiveness insofar as professionals specialized in tumor pathologies (eg, esophageal cancer), organ systems (eg, gynecological tumors), or patient profiles (eg, pediatric oncology) are responsible for clinical coordination and communication with patients and families throughout all stages of the disease. To this end, the AEMAC program can provide a comprehensive reflection on the organizational, technological, and cultural elements that must be taken into account to improve the care received by patients with cancer.

## Appendix

Multimedia Appendix 1 Supplementary data.

Multimedia Appendix 2 Phase 1: Individual self-assessment.

Multimedia Appendix 3 Phase 2: Group prioritization process.

Multimedia Appendix 4 Phase 3: Creating an improvement plan.

Multimedia Appendix 5 Experience survey to assess critical issues of the Autoevaluación de Equipos Multidisciplinares de Atención al Cáncer program.

## Abbreviations

AEMAC: Autoevaluación de Equipos Multidisciplinares de Atención al Cáncer
MDT: multidisciplinary team
MDT-FIT: multidisciplinary team-feedback for improving teamworking
MDT-MODe: multidisciplinary team-metric of decision making
MDT-OARS: multidisciplinary team-observational assessment rating scale
MDT-QuIC: multidisciplinary team-quality improvement checklist
SNS: Spanish National Health System
