# Supplementary material for: A Web-Based Self-assessment Model for Evaluating Multidisciplinary Cancer Teams in Spain: Development and Validation Pilot Study
Source: J Med Internet Res. 2022 Mar 10;24(3):e29063. doi: 10.2196/29063 (PMC8949680; doi:10.2196/29063)
Supplement: Multimedia Appendix 1 [file jmir_v24i3e29063_app1.docx]

Supplementary material.

Table S1. Scientific societies involved in the AEMAC Program.

| Scientific societies | | |
| --- | --- | --- |
| Spanish Association of Surgeons (AEC) | Spanish Society of Medical Oncology (SEOM) | Spanish Society of Radiation Oncology (SEOR) |
| Spanish Society of Medical Radiology (SERAM) | Spanish Society of Pediatric Hematology and Oncology (SEHOP) | Spanish Society of Hospital Pharmacy (SEFH) |
| Spanish Society of Hematology and Hemotherapy (SEHH) | Spanish Society for Healthcare Quality (SECA) | Spanish Society of Pathological Anatomy (SeAP-IAP) |
| Spanish Oncology Nursing Society (SEEO) | | |

Table S2. Thematic areas and components of the AEMAC Program for self-assessment of multidisciplinary care.

| **First thematic area: PREPARATION AND ORGANIZATION OF TUMOR BOARDS** | | |
| --- | --- | --- |
| **1. Attendance and representation.** The core specialties are pathology, radiology, surgery, medical oncology, radiation oncology (if available at the center or included through an inter-hospital agreement), hematology, specialties of the specific tumor field (pulmonology, urology, gastroenterology, gynecology, etc.) and nursing [1]. Other specialists can attend as needed [2] (geriatrician, plastic surgeon, palliative care specialist, etc.). | | |
| Professionals from diagnostic, medical and surgical specialties participate, but certain problems (e.g. organizational, scheduling) often limit the attendance of one or more specialties. | Professionals from all core diagnostic and therapeutic specialties attend [1], including nursing staff. | Professionals from all core diagnostic and therapeutic specialties attend [1], including nursing staff and other specialists as needed [2]. |
| **2. Patient schedule.** The Board members are provided with the patient agenda in advance of the meeting, which facilitates the preparation of all tests and clinical/non-clinical information for each case. Nonetheless, the board is open to new patients who, due to healthcare needs, must be included without notice. | | |
| There is no agenda as such, each specialist discusses their patients at the board meeting. | The agenda remains open until the time of the meeting, limiting the possibility of preparing and having all the relevant information on each patient in advance. | The agenda is completed and made available to all board members before the meeting so that tests and relevant information on each patient can be prepared in advance. |
| **3. Meeting frequency.** The board meeting is held periodically according to an established schedule as part of the whole healthcare process. | | |
| Monthly meeting, or according to volume of cases | Meeting every 10 or 15 days | Weekly meeting |
| **4. Cases discussed.** All cases treated in the hospital for a specific pathology are presented at the board meeting. | | |
| Complex cases that fall outside the established protocol are discussed. | All potentially complex cases (50%-80%) are discussed, including recurrences. | Between 80% and 100% of cases are discussed, including recurrences. |
| **5. Involvement of the professionals responsible for the patients.** Professionals present their cases to the board and are involved in the decision-making process regarding their patients. In this way, they can explain the therapeutic proposal and weigh up alternatives that may have arisen in the discussion. | | |
| Professionals are involved in the board decision-making process regarding 10%-50% of their patients. | Professionals are involved in the board decision-making process regarding 50%-80% of their patients. | Professionals are involved in the board decision-making process regarding 80%-100% of their patients. |
| **6. Time management.** The following criteria must be met to ensure efficient management of the board’s time: (1) the scheduled time slot for the meeting is respected; (2) the information on the cases to be discussed is presented in a structured way (diagnostic and staging data, comorbidities, psychosocial aspects, etc.); (3) the whole team follows the same multidisciplinary clinical protocol or clinical practice guide (CPG); (4) all cases of the pathology are presented, although for those considered straightforward the decision is simply validated. | | |
| 1 or 2 criteria are satisfied. | 3 criteria are satisfied. | All 4 criteria are satisfied. |
| **7. Case presentation.** Cases are presented and discussed on the board prior to any treatment planning. Treatment plans are determined jointly on the board. | | |
| Treatment plans are determined by the board in 10%-50% of cases. | Treatment plans are determined by the board in 50%-80% of cases. | Treatment plans are determined by the board in 80%-100% of cases. |
| **Second thematic area: TUMOR BOARD DECISION-MAKING PROCESS** | | |
| **8. Learning and updating knowledge.** The board is responsible for preparing and updating the multidisciplinary clinical protocol and discussing new scientific evidence. Part of the board time is devoted to these aims, or separate multidisciplinary scientific sessions are held for this purpose. | | |
| The board does not allocate a specific time to discussing new scientific evidence; individual departments may need to perform this task. | The board is responsible for updating the multidisciplinary clinical protocol. | The board is responsible for updating the multidisciplinary clinical protocol and discussing new evidence during specific sessions. |
| **9. Psychosocial perspective.** The multidisciplinary team takes into consideration the psychosocial context of each patient (e.g. independence, health and hygiene, family support) insofar as these factors can affect planning and adherence to treatment. | | |
| The psychosocial context of each patient is not considered during case discussion. | The psychosocial context is considered informally and spontaneously during the discussion of cases on the board. | Guidelines have been established for detecting psychosocial problems that may be relevant in treatment planning, and these guidelines are followed during the board discussion. |
| **10. Oncogeriatrics perspective.** The multidisciplinary team includes the oncogeriatric perspective in the decision-making process to enable adjustment of therapeutic planning and thus improve quality of care for older patients. A professional with specific responsibilities assesses frailty on an established scale to facilitate a subsequent comprehensive geriatric assessment. | | |
| The patient is assessed according to the usual clinical parameters, which do not include the oncogeriatric perspective. | A specific professional assesses frailty on an established scale and the board includes this variable in the decision-making process. | There is an action protocol in place that includes evaluation of frailty on an established scale by a specific professional, a comprehensive geriatric assessment, and adjusted decision-making by the multidisciplinary team. |
| **11. Clinical trials.** Board members share information on open clinical trials and patient profiles to facilitate the inclusion of candidate patients. If the hospital is not participating in a given trial, the patient can be referred to another hospital for inclusion. | | |
| Open clinical trials (in any hospital) are not discussed on the board as they concern the corresponding specialties. | The possibility of certain patients participating in a specific clinical trial (in the same hospital or through interhospital referral) is evaluated by the board. | The board systematically reports on open clinical trials and encourages patient inclusion (in the same hospital or through interhospital referral). |
| **12. Patient information process.** The patient information process related to the treatment plan involves reporting and sharing information on: (1) benefits and risks associated with each therapeutic option (if there is more than one) according to the desired level of information; (2) possible side effects (acute and chronic); (3) possible late effects (as a consequence of treatment, e.g. cardiac alterations); (4) healthy lifestyles (e.g. physical exercise, nutrition, and sexual and psychological wellbeing) as part of the therapeutic plan to improve health and help prevent recurrences or second malignant neoplasms (where relevant). | | |
| 1 or 2 criteria are satisfied. | 3 criteria are satisfied. | All 4 criteria are satisfied. |
| **Third thematic area: CONTINUITY OF CARE** | | |
| **13.** **Computerized record of decisions.** The cases discussed and decisions taken on the board are recorded for subsequent access on the computerized medical record. | | |
| Board decisions are not recorded. All professionals take their own notes. | Board decisions are recorded but are not accessible through computerized medical records. | Board decisions are recorded and are accessible through computerized medical records. |
| **14. Decision implementation.** The decisions taken by the board are binding and are implemented unless changes in the patient's health prevent this action. Professionals who do not implement decisions must justify this departure from protocol to the board. | | |
| Less than 50% of the decisions taken on the board are implemented. | Between 50% and 80% of the decisions taken on the board are implemented. | More than 80% of the decisions taken on the board are implemented. |
| **15. Follow-up planning.** Coordinating patient follow-up ensures that a single professional (or 2, if different assessments are required) is responsible for conducting follow-up visits, respecting the tests and control intervals for the disease and side effects of the treatments. Because visits are not duplicated, the burden of care is reduced and resources are optimized. | | |
| The follow-up of patients is not coordinated by the team. Professionals from different services carry out their own follow-up visits. | The team coordinates patient follow-up so that a single professional (or 2, if different assessments are required) is responsible for follow-up visits. | The team coordinates patient follow-up so that a single professional (or 2, if different assessments are required) is responsible for follow-up visits. Follow-up planning is included in the clinical protocol for the pathology. |
| **16. Care for long-term survivors of cancer.** After the follow-up stage, when patients can be considered long-term cancer survivors according to their clinical status (e.g. at 5 years), they are provided with: (1) personalized information on managing chronic side effects and on the possible appearance of late effects (as a consequence of the treatments received, e.g. cardiac alterations); (2) continuation of oncological follow-up in primary care as part of a coordinated and formalized transition between the 2 levels of care. | | |
| No criteria are satisfied. | 1 criterion is satisfied. | Both criteria are satisfied. |
| **Fourth thematic area: ORGANIZATIONAL CONTEXT OF THE TEAMS** | | |
| **17. Board time protection**. Department heads protect the time slot allocated to the session and at hospital management level, board time is recognized as clinical time in all care processes. The protection of board time is considered essential for the burden of care to be compatible with participation. | | |
| Board time is unprotected, which can lead to attendance problems for some members. | Department heads protect board time to facilitate the attendance of the different members. | Department heads protect board time and hospital management recognizes the activity of the multidisciplinary teams as a healthcare device, which normalizes participation. |
| **18. Administrative support.** The board has specific administrative support for managing all multidisciplinary activity before, during and after the board meeting (e.g. to summon professionals, update patient lists, draw up the minutes, and manage new appointments), which ensures streamlined care coordination and team organization. | | |
| The board does not have administrative support. | The team members themselves take on the administrative tasks arising from the multidisciplinary activity. | The board has specific administrative staff for the procedures arising from the multidisciplinary activity. |
| **19. Meeting room.** The board meetings are held in a physical space that (1) has good light; (2) is comfortable (including enough chairs and tables); (3) is free of interruptions; (4) has good acoustics; (5) is easily accessible and close to members’ place of work; (6) ensures ease of interaction. | | |
| 1 or 2 criteria are satisfied. | 3 or 4 criteria are satisfied. | 5 or all 6 criteria are satisfied. |
| **20. Technological resources.** The board has access to the following technological resources: (1) at least 2 computers in the room to record on site all decisions taken and all queries made; (2) a high-definition projector for viewing images; (3) a double screen; (4) technology enabling real-time consultation of computerized medical histories; and (5) technology enabling videoconferences. | | |
| 1 or 2 criteria are satisfied. | 3 or 4 criteria are satisfied. | 5 criteria are satisfied. |
| **21. Role of the hospital tumor board (HTB).** The hospital tumor board (HTB) facilitates the establishment of quality objectives and indicators for the oncology field at the hospital level, as well as facilitating dialogue with the management and homogenization of multidisciplinary teams based on the available resources and good practices. | | |
| The HTB is not a relevant actor for multidisciplinary care in the hospital. | The HTB is a relevant actor that establishes quality objectives and indicators for all multidisciplinary teams. | The HTB is a relevant actor that establishes quality objectives and indicators for all multidisciplinary teams, as well as helping to standardize the resources and organizational practices of the different teams. |
| **Fifth thematic area: CROSS-DISCIPLINARY ROLES AND TEAM COHESION** | | |
| **22. Board chair or coordinator.** The coordinator or chair ensures that all members play an independent role on the board, actively facilitating effective and efficient decision-making. They perform organizational functions (e.g. ensuring attendance) and promote the scientific development and talent of the team (e.g. by organizing scientific sessions). The coordinator has institutional recognition and acts as the interlocutor between the different departments and medical management to improve quality of care (e.g., highlighting the need for resources). | | |
| The coordinator does not actively influence the dynamics of the board or perform organizational functions. | The coordinator facilitates consensus and helps bring the team together. This person performs organizational functions and contributes to promoting the scientific development of the team. | The coordinator facilitates consensus and helps bring the team together, as well as performing organizational functions and helping to promote the scientific development of the team. This person has institutional recognition and acts as the interlocutor with the medical management level. |
| **23.** **Nursing case manager**. The nursing case manager helps to organize the entire care process and performs a coordinating role within the multidisciplinary team, as well as offering personalized care to patients (e.g. speeding up tests, organizing agendas, ensuring patients understand the clinical information received) and guaranteeing continuity of care. Case management presupposes expert clinical knowledge of one or more specific pathologies. | | |
| A nursing case manager has not been formally appointed. | A nursing case manager has been appointed only part time, or their participation is limited by professional requirements (e.g. scheduling problems limit board meeting attendance), which hinders their professional development in this role. | The board has a formal nursing case manager with autonomy and expert clinical knowledge. |
| **24. Team cohesion.** A cohesive team meets the following criteria: (1) mutual respect; (2) fluid and informal communication; (3) equal evaluation of all clinical and other opinions; (4) active participation of all members, welcoming individual opinions; (5) common goals and expectations; (6) psychological well-being and satisfaction derived from participation and integration in the team. | | |
| 1 or 2 criteria are satisfied. | 3 or 4 criteria are satisfied. | 5 or all 6 criteria are satisfied. |
| **25. Team-patient communication framework.** Communication with the patient is understood to be a consistent process with different stages. It must meet the following criteria: (1) the information that the patient is able to and wishes to understand, is adapted for this purpose; (2) patients can ask questions at all times during the care process; (3) there is a clear division of communicative responsibilities (e.g. the radiologist can confirm the diagnosis to the patient, or not) to prevent disparity in the information shared (e.g. extent of the disease, treatment plan, etc.); (4) patients participate in therapeutic decision-making; (5) information shared with the patient is recorded in their medical record (e.g. prognosis) | | |
| 1 or 2 criteria are satisfied. | 3 or 4 criteria are satisfied. | All 5 criteria are satisfied. |
| **26. Key points in team-patient communication.** There are 4 events during the care process in which team-patient communication must be carefully managed to prevent emotional distress for patients. The criteria relating to these events are as follows: (1) the diagnosis is shared openly and with empathy; (2) clinicians transmit tranquility and security at the patient’s last visit following completion of treatment, bearing in mind many patients feel sad and uncertain at this time; (3) recurrence is considered a new process and the oncology team takes into account that the patient’s personal and emotional situation may have changed since the previous diagnosis; (4) the progress of the disease is explained clearly, managing the patient’s expectations and needs. | | |
| 1 or 2 criteria are satisfied. | 3 criteria are satisfied. | All 4 criteria are satisfied. |
| **27. Team evaluation.** By evaluating processes and clinical results, the team can measure quality of care. Having common indicators and activity data facilitates regular discussion (e.g. annually) about the results and possible future changes. Ideally, a shared database is in place to gather indicators such as time intervals in the care process, tumor staging, level of adherence to the clinical protocol, and toxicities. | | |
| The responsibilities of the board do not include evaluating results. | The clinical data generated by a specialist or department (e.g., for publication) are used to discuss aspects of quality of care on the board. | Information systems are in place to record quality indicators of the pathology, and the results are discussed regularly on the board. |

Table S3. Frequencies for each component. N = 243

| Aspect | Component |  | Frequency | Percentage |
| --- | --- | --- | --- | --- |
| Preparation and Organization of the Board | Attendance and representation | Segment 1 | 144 | 59.3 |
|  |  | Segment 2 | 23 | 9.5 |
|  |  | Segment 3 | 76 | 31.3 |
|  | Patient schedule | Segment 1 | 15 | 6.2 |
|  |  | Segment 2 | 79 | 32.5 |
|  |  | Segment 3 | 149 | 61.3 |
|  | Meeting frequency | Segment 1 | 10 | 4.1 |
|  |  | Segment 2 | 68 | 28 |
|  |  | Segment 3 | 165 | 67.9 |
|  | Cases discussed | Segment 1 | 43 | 17.7 |
|  |  | Segment 2 | 84 | 34.6 |
|  |  | Segment 3 | 116 | 47.7 |
|  | Involvement of the professionals responsible for the patients | Segment 1 | - | - |
|  |  | Segment 2 | 56 | 23 |
|  |  | Segment 3 | 187 | 77 |
|  | Time management efficiency | Segment 1 | 58 | 23.9 |
|  |  | Segment 2 | 79 | 32.5 |
|  |  | Segment 3 | 106 | 43.6 |
|  | Case presentation | Segment 1 | 15 | 6.2 |
|  |  | Segment 2 | 85 | 35 |
|  |  | Segment 3 | 143 | 58.8 |
| Board decision-making process | Learning and updating knowledge | Segment 1 | 102 | 42 |
|  |  | Segment 2 | 57 | 23.5 |
|  |  | Segment 3 | 84 | 34.6 |
|  | Psychosocial perspective | Segment 1 | 15 | 6.2 |
|  |  | Segment 2 | 194 | 79.8 |
|  |  | Segment 3 | 34 | 14 |
|  | Oncogeriatrics perspective | Segment 1 | 165 | 67.9 |
|  |  | Segment 2 | 40 | 16.5 |
|  |  | Segment 3 | 38 | 15.6 |
|  | Clinical trials | Segment 1 | 82 | 33.7 |
|  |  | Segment 2 | 117 | 48.1 |
|  |  | Segment 3 | 44 | 18.1 |
|  | Patient information process | Segment 1 | 17 | 7 |
|  |  | Segment 2 | 84 | 34.6 |
|  |  | Segment 3 | 142 | 58.4 |
| Continuation of the care process | Computerized record of decisions | Segment 1 | 6 | 2.5 |
|  |  | Segment 2 | 10 | 4.1 |
|  |  | Segment 3 | 227 | 93.4 |
|  | Decision implementation | Segment 1 | - | - |
|  |  | Segment 2 | 34 | 14 |
|  |  | Segment 3 | 209 | 86 |
|  | Follow-up planning | Segment 1 | 60 | 24.7 |
|  |  | Segment 2 | 86 | 35.4 |
|  |  | Segment 3 | 97 | 39.9 |
|  | Care for long-term survivors of cancer | Segment 1 | 43 | 17.7 |
|  |  | Segment 2 | 115 | 47.3 |
|  |  | Segment 3 | 85 | 35 |
| Organizational Context | Board time protection | Segment 1 | 102 | 42 |
|  |  | Segment 2 | 43 | 17.7 |
|  |  | Segment 3 | 98 | 40.3 |
|  | Administrative support | Segment 1 | 119 | 49 |
|  |  | Segment 2 | 93 | 38.3 |
|  |  | Segment 3 | 31 | 12.8 |
|  | Meeting room | Segment 1 | 28 | 11.5 |
|  |  | Segment 2 | 73 | 30 |
|  |  | Segment 3 | 142 | 58.4 |
|  | Technological resources | Segment 1 | 141 | 58 |
|  |  | Segment 2 | 88 | 36.2 |
|  |  | Segment 3 | 14 | 5.8 |
|  | Role of the Hospital Tumor Board | Segment 1 | 78 | 32.1 |
|  |  | Segment 2 | 117 | 48.1 |
|  |  | Segment 3 | 48 | 19.8 |
| Cross-disciplinary roles and team cohesion | Board chair and coordinator | Segment 1 | 30 | 12.3 |
|  |  | Segment 2 | 114 | 46.9 |
|  |  | Segment 3 | 99 | 40.7 |
|  | Nursing case manager | Segment 1 | 187 | 12.3 |
|  |  | Segment 2 | 21 | 46.9 |
|  |  | Segment 3 | 35 | 40.7 |
|  | Team cohesion | Segment 1 | 11 | 4.5 |
|  |  | Segment 2 | 60 | 24.7 |
|  |  | Segment 3 | 172 | 70.8 |
|  | Team-patient communication framework | Segment 1 | 16 | 6.6 |
|  |  | Segment 2 | 109 | 44.9 |
|  |  | Segment 3 | 118 | 48.6 |
|  | Key moments in team-patient communication | Segment 1 | 22 | 9.1 |
|  |  | Segment 2 | 66 | 27.2 |
|  |  | Segment 3 | 155 | 63.8 |
|  | Team evaluation | Segment 1 | 146 | 60.1 |
|  |  | Segment 2 | 71 | 29.2 |
|  |  | Segment 3 | 26 | 10.7 |
