# Supplementary figures and images for: A Web-Based Self-assessment Model for Evaluating Multidisciplinary Cancer Teams in Spain: Development and Validation Pilot Study
Source: J Med Internet Res. 2022 Mar 10;24(3):e29063. doi: 10.2196/29063 (PMC8949680; doi:10.2196/29063)

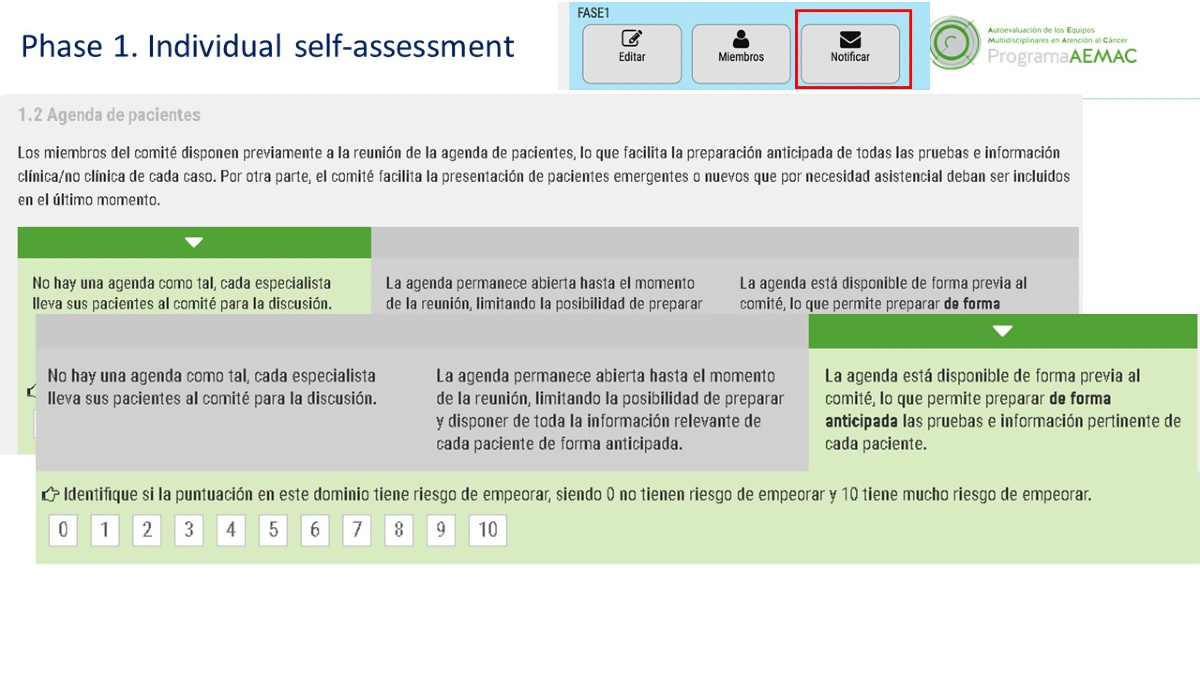

Supplement: Multimedia Appendix 2 [file jmir_v24i3e29063_app2.png]

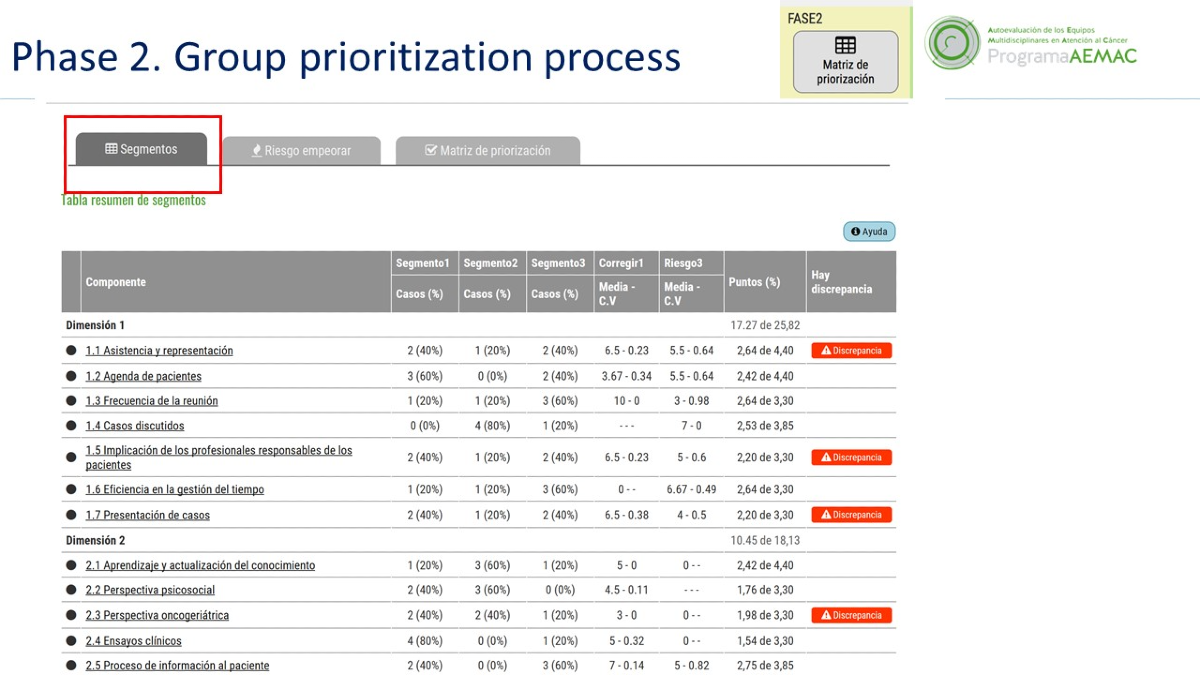

Supplement: Multimedia Appendix 3 [file jmir_v24i3e29063_app3.png]

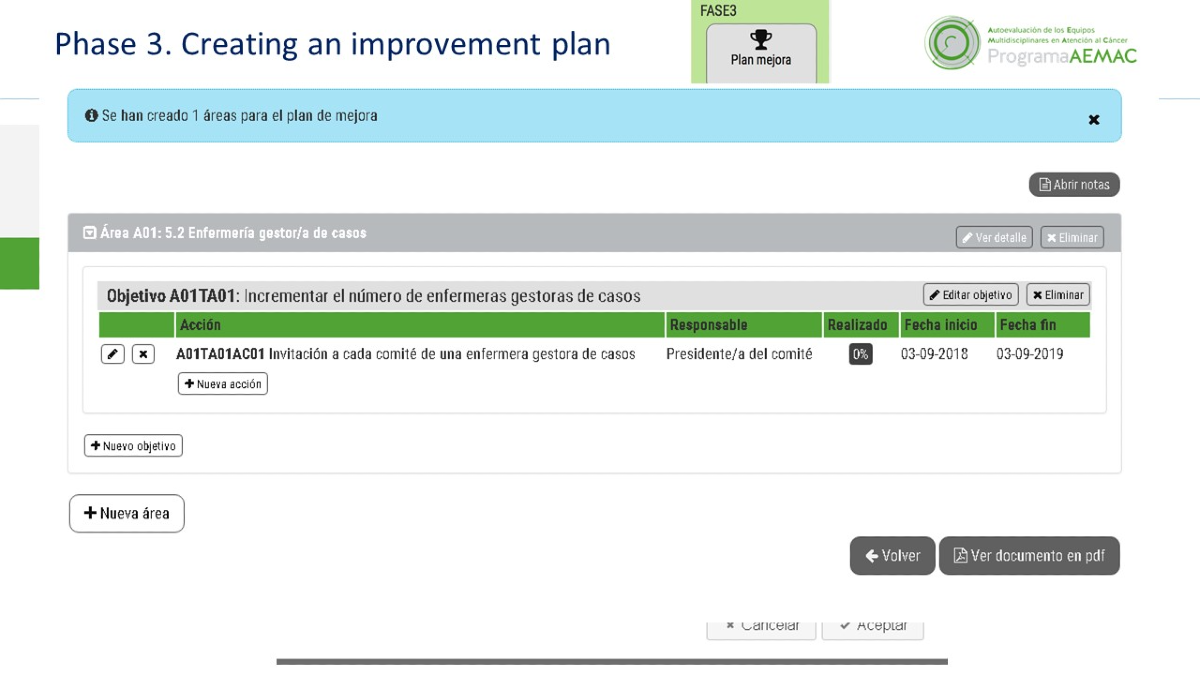

Supplement: Multimedia Appendix 4 [file jmir_v24i3e29063_app4.png]
