# Supplementary material for: A Web-Based Self-assessment Model for Evaluating Multidisciplinary Cancer Teams in Spain: Development and Validation Pilot Study
Source: J Med Internet Res. 2022 Mar 10;24(3):e29063. doi: 10.2196/29063 (PMC8949680; doi:10.2196/29063)
Supplement: Multimedia Appendix 5 [file jmir_v24i3e29063_app5.docx]

**Multimedia Appendix 5. Experience survey to assess critical issues of the AEMAC PROGRAM**

**Thank you for your collaboration in the pilot test of the AEMAC Programme.**

**Please rate (0 to 10) the following points according to your user experience:**

|  | 0 a 10 |
| --- | --- |
| Ease of answering the questions |  |
| Coherence between content of questions and reality of tumor boards |  |
| Adequacy of the 3-option response scale |  |
| Ease of creating an improvement plan on the online application |  |
| Overall satisfaction with the self-assessment |  |

**Please rate (mark X) as adequate or non adequate the selection of components considered as critical (especially relevant, essential, to ensure a proper functioning of the Committees):**

| **Critical components** | **Adequate** | **Non adequate** |
| --- | --- | --- |
| 1.1 Attendance and representation |  |  |
| 1.2 Patient schedule |  |  |
| 2.1 Learning and updating knowledge |  |  |
| 3.2 Decision implementation |  |  |
| 3.3 Follow-up planning |  |  |
| 4.1 Board time protection |  |  |
| 5.1 Board chair or coordinator |  |  |
| 5.2 Nursing case manager |  |  |

**Please rate (mark X) as adequate or non adequate the selection of components considered as critical (especially relevant, essential, to ensure a proper functioning of the Committees):**

| **Semi-critical components** | Acertada | Desacertada |
| --- | --- | --- |
| 1.4 Cases discussed |  |  |
| 2.5 Patient information process |  |  |
| 3.1 Computerized record of decisions |  |  |
| 5.5 Key points in team-patient communication |  |  |

Please indicate in this space if you would like to make any suggestions that could improve the functioning of the Self-Assessment session:

**Thank you very much for your cooperation**
